# Semi-Mechanism-Based Pharmacokinetic-Toxicodynamic Model of Oxaliplatin-Induced Acute and Chronic Neuropathy

**DOI:** 10.3390/pharmaceutics12020125

**Published:** 2020-02-03

**Authors:** Shinji Kobuchi, Risa Shimizu, Yukako Ito

**Affiliations:** Department of Pharmacokinetics, Kyoto Pharmaceutical University, Kyoto 607-8414, Japan; kobuchi@mb.kyoto-phu.ac.jp (S.K.);

**Keywords:** pharmacokinetic-toxicodynamic modeling, peripheral neuropathy, cancer chemotherapy, platinum compound, personalized therapy, toxicology

## Abstract

Oxaliplatin (L-OHP) is widely prescribed for treating gastroenterological cancer. L-OHP-induced peripheral neuropathy is a critical toxic effect that limits the dosage of L-OHP. An ideal chemotherapeutic strategy that does not result in severe peripheral neuropathy but confers high anticancer efficacy has not been established. To establish an optimal evidence-based dosing regimen, a pharmacokinetic-toxicodynamic (PK-TD) model that can characterize the relationship between drug administration regimen and L-OHP-induced peripheral neuropathy is required. We developed a PK-TD model of L-OHP for peripheral neuropathy using Phoenix^®^ NLME™ Version 8.1. Plasma concentration of L-OHP, the number of withdrawal responses in the acetone test, and the threshold value in the von Frey test following 3, 5, or 8 mg/kg L-OHP administration were used. The PK-TD model consisting of an indirect response model and a transit compartment model adequately described and simulated time-course alterations of onset and grade of L-OHP-induced cold and mechanical allodynia. The results of model analysis suggested that individual fluctuation of plasma L-OHP concentration might be a more important factor for individual variability of neuropathy than cell sensitivity to L-OHP. The current PK-TD model might contribute to investigation and establishment of an optimal dosing strategy that can reduce L-OHP-induced neuropathy.

## 1. Introduction

The platinum (Pt)-based anticancer agent oxaliplatin (L-OHP) is a key drug used for the treatment of advanced or metastatic colorectal cancer in some chemotherapy regimens, including CapOX (L-OHP and capecitabine), SOX (L-OHP and S-1), and FOLFOX (L-OHP and 5-fluorouracil/leucovorin) [1,2,3,4]. The dosing schedule of each regimen was as follows: CapOX and SOX regimen: 2 h infusion of 130 mg/m^2^ L-OHP, every three weeks as one cycle, FOLFOX regimen: 2 h infusion of 85–130 mg/m^2^ L-OHP, every two weeks as one cycle [2,3,4]. Preclinical studies have focused on the pharmacokinetic-toxicodynamic relationships to predict the toxicity of L-OHP [5,6]. Despite favorable antitumor effects of L-OHP, undesirable adverse effects such as nausea, vomiting, myelosuppression, and peripheral neuropathy contribute to treatment failure. Unlike other antitumor agents, acute and chronic peripheral neuropathy are well-known frequent and serious toxic effects of L-OHP, resulting in discontinuation of chemotherapy and poor prognosis [7,8]. Therefore, a challenge to improving clinical outcomes in L-OHP-based chemotherapy is to elucidate mechanisms of drug-induced peripheral neuropathy and to develop alternative dosing strategies that do not induce severe peripheral neuropathy.

Acute neuropathy is specific to L-OHP and occurs in approximately 90% of patients within hours after administration [9]. This neuropathy is characterized by cold hypersensitivity, throat discomfort, and dysesthesia of the peripheral regions including hand, foot, and perioral regions, affecting the patients’ daily activities. Alteration of voltage-gated Na^+^ and K^+^ channels has been reported as the major mechanism of these effects [10,11,12,13]. In addition, continued exposure to L-OHP also induces chronic neuropathy in approximately 70% of the patients, causing temperature-insensitive paresthesia, hypoesthesia, and dysesthesias of the hands and feet [14,15]. To reduce L-OHP-induced acute neuropathy, calcium and magnesium infusions [16,17] and gabapentin administration [18,19] are recommended. However, these treatments do not provide adequate clinical benefit [20], and the chronic neuropathy remains as a dose-limiting side effect [21,22].

Recently, we performed pharmacokinetic (PK) and toxicodynamic (TD) studies of L-OHP in rats [23]. We demonstrated that cold and mechanical allodynia occurred more frequently and more intensely with increased L-OHP doses. These results indicated that dosage regulation might be an attractive strategy to prevent neurotoxic effects that limit L-OHP clinical efficacy. However, pharmacokinetic-toxicodynamic (PK-TD) modeling based on PK and TD data is required to link L-OHP exposure to peripheral neuropathy and allow for the development of evidence-based dosing strategies [23]. A mathematical approach such as the use of a PK-TD model is a feasible alternative because it can link the drug dosing regimen to drug toxicities and some PK-TD models of anticancer agents using preclinical data have been reported [24,25,26].

The aim of the current study was to develop a PK-TD model to predict time course and degree of L-OHP-induced acute and chronic neuropathy and to simulate the influence of alternative dosing on neuropathy.

## 2. Materials and Methods

### 2.1. PK and TD Data Source

Individual plasma L-OHP concentration (μg/mL) and quantitative TD data of the acetone test (the unit: times) and the von Frey filament test (the unit: g) reported in our previous study [23] with increased sample size were used as the source of data for PK-TD modeling in the present study. Animal protocols in our previous study were approved by the Institutional Animal Care and Use Committee (Permit number: PKPD-16-001, Date of approval: 26 April 2016), and the studies were performed in accordance with the Guidelines for Animal Experimentation of Kyoto Pharmaceutical University. PK and TD data were obtained as described below.

In the PK study, 10-week-old male Wistar rats (weighing 300–360 g, *n* = 15) were divided randomly into three groups based on L-OHP dose administered. Rats in each group were administered 3, 5, or 8 mg/kg L-OHP (Elplat^®^ [YakultHonsha Co., Ltd., Tokyo, Japan], 5 mg/mL) into the external right jugular vein. The L-OHP dose was determined based on clinical doses and previous animal studies investigating L-OHP toxicity [6,27]. Blood samples (0.25 mL) were withdrawn from the right jugular vein at 3, 5, 10, 20, 30, 45, 60, 90, and 120 min after L-OHP administration. Due to unstable L-OHP in the presence of plasma protein, the obtained blood sample was immediately centrifugated and 100 μL plasma sample was deprotenized by 200 μL acetonitrile. Plasma concentration of L-OHP was determined by liquid chromatography-tandem mass spectrometry (LC-MS/MS) according to the method reported by Minakata et al. [28]. The measured values of Pt derivative were used for L-OHP concentration.

In the TD study, L-OHP-induced cold and mechanical allodynia were assessed by the acetone test [29] and the von Frey filament test [30], respectively. All rats (*n* = 21) were divided randomly into four groups based on the control and L-OHP doses administered. Rats in each group were administered intravenous saline, or 3, 5, or 8 mg/kg L-OHP (5 mg/mL) once per week for four weeks. In the acetone test, the number of withdrawal responses was counted for 40 s after 50 μL of acetone was sprayed onto the plantar skin. The test was conducted six times (three times per hind limb) and the mean number of withdrawal responses was calculated from the ratio of the number of total responses to six. This mean value was used for assessing acute neuropathy. In the von Frey filament test, the withdrawal response was observed after von Frey filaments (Touch Test^®^ Sensory Evaluator, North Coast Medical, Inc., Gilroy, CA, USA) were applied to each hind paw. The threshold value was determined by the weakest stimulation that caused a positive response according to the up-down method of the von Frey filament test. These TD evaluations were conducted three days per week (Monday, Wednesday, and Friday) after dosing. The number of withdrawal responses and the threshold value were used to develop PK-TD models of acute and chronic neuropathy model. Further details of these methods have been described in our previous report [23].

### 2.2. PK-TD Model Development

#### 2.2.1. Software and Criteria

**Figure 1:**
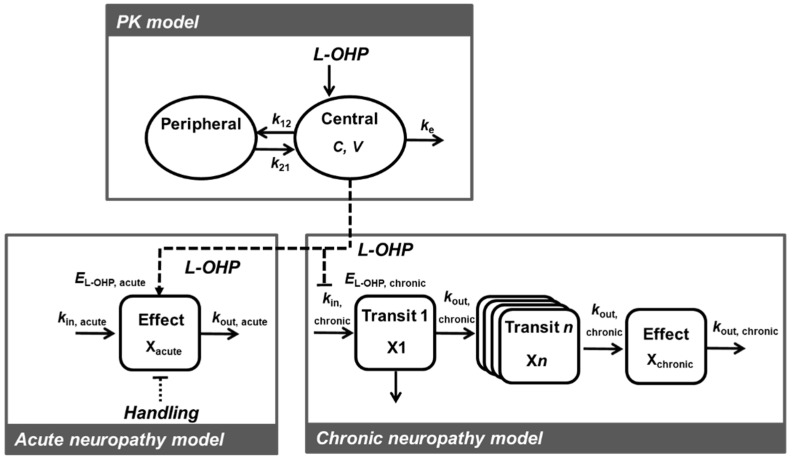
Schematic of the pharmacokinetic-toxicodynamic (PK-TD) model of oxaliplatin (L-OHP) induced acute and chronic neuropathy. C, L-OHP concentration in the central compartment; *V*, central volume of the distribution; *k_e_*, elimination rate constant from the central compartment; *k*_12_, rate constant of central compartment to peripheral compartment; *k*_21_, rate constant of peripheral compartment to central compartment; *E_L-OHP,acute_*, drug effect for acute neuropathy; *E_handling_*, handling effect; *k_in,acute_*, a zero-order rate constant describing the rate of increase of paw withdrawal responses; *k_out,acute_*, a first-order rate constant describing the rate of decrease of paw withdrawal responses; *E_L-OHP,chronic_*, drug effect for chronic neuropathy; *k_in chronic_*, a zero-order rate constant describing the rate of increase of paw withdrawal threshold; *k_out,chronic_*, a first-order rate constant describing the rate of decrease of paw withdrawal threshold.

PK-TD models to describe time course alterations of plasma concentrations of L-OHP and onset and degree of L-OHP-induced neuropathy were developed using a non-linear mixed effects modeling program—Phoenix^®^ NLME™ Version 8.1 software (Certara USA, Inc., Princeton, NJ, USA). The PK-TD model was developed using a two-stage approach. PK model development and parameters were estimated separately from the TD model, and each PK parameter was fixed when developing the TD model. To estimate model parameters and their variability, first-order conditional estimation with the extended least squares method was used. Structural model selection was based on Akaike’s Information Criteria (AIC), goodness-of-fit plots including population predictions (PRED) vs. observations (OBS), individual predictions (IPRED) vs. OBS, conditional weighed residuals (CWRES) vs. time, CWRES vs. IPRED, and the coefficient of variation (CV) of parameter estimates. For non-nested models, AIC value was used for selection between models. A drop in AIC of two or more was applied as the cutoff criterion for PK and TD model improvement, which was the threshold for selecting one model over another [31]. For nested models, the minimum value of −2 × log likelihood (−2LL), served as a guide during model building. A decrease in −2LL of 6.63 points for an added parameter was regarded as a significant improvement of the model. A schematic of the final PK-TD model is shown in Figure 1.

#### 2.2.2. PK Model

The PK model was chosen based on previous studies [32,33] and the experimental data. Only plasma L-OHP concentration data following single administration were used for PK modeling, whereas we have also these data following multiple administration. In our previous report [23], after the fourth administration of L-OHP, higher plasma L-OHP levels were observed. Although a suspected mechanism of this phenomenon is the decrease in renal excretion of the drug by multiple doses-induced renal failure, the details are still unknown to develop the appropriate PK model. Moreover, it is difficult to estimate the timing of this increase in plasma concentration in the dosing regimen for four weeks. Therefore, to be more concise, only plasma L-OHP concentration data on Day 1 were used for PK modeling. The linear elimination model was adapted because the results of in vivo study shows dose proportional increase in AUC and unchanged half-life of L-OHP with increasing dose [23]. Two-compartment models with linear elimination was applied to describe the course of the L-OHP plasma concentration levels according to the previous report [32]. Different inter-individual variability models (the exponential and additive) were initially tested. Inter-individual variability of PK parameters was characterized by the exponential error model. Based on the above-mentioned criteria, the inter-individual variability model was used for *V*, *k_e_*, *k*_12_, and *k*_21_. Different residual error models (including the additive, proportional, combined or power) were tested and residual variability in measurements of C was assumed using a proportional error model. The diagnosis plots of PK model are shown in Figure S1.

#### 2.2.3. TD Model for Acute Neuropathy

Habituation was observed in the control group, suggesting that animal handling affected the number of paw withdrawal responses in the acetone test. In previous toxicological studies using rats, body weight loss was observed due to animal handling and this effect was considered in development of the TD model [33,34,35]. To characterize the effects of animal handling on the number of paw responses, the handling effects was simply added to the response model as following equations:

(1) $$x_{acute}\left(t\right)=Response\left(t\right)+Handling\left(t\right),$$

(2) $$Handling\left(t\right)=\;x_{0,\;acute}\cdot \;e^{-k_{handling}\cdot t},$$

(3) $$x_{acute}\left(0\right)=Handling\left(0\right)=\;x_{0,\;acute},$$

where *x_acute_*(*t*) was the number of paw withdrawal responses, *Response*(*t*) was the drug effects on the number of paw responses, *Handling*(*t*) was the effects of animal handling on the number of paw responses, *k_handling_* was the constant in the handling effect, *x*_0,*acute*_ was the baseline value of the responses. Based on the observed data in the control group, the baseline value of the responses was 3.1 times. The observed data in control group was preliminary fitted with this model and the value *k_handling_* = 0.08 allowed the TD model to effectively capture the features of TD data. Then, the value of *x*_0,*acute*_ and *k_handling_* was fixed at 3.1 and 0.08, respectively.

For PK-TD analysis of acetone test data in L-OHP treated rats, both animal handling and the drug affected the number of paw withdrawal responses. The linear, *E_max_*, or sigmoid-*E_max_* models were considered to describe time course alterations data. L-OHP was assumed to increase the rate of paw withdrawal responses as described by the sigmoid-*E_max_* model and the following equations:

(4) $$\frac{dResponse\left(t\right)}{dt}=k_{in,\;acute}+E_{L-OHP,acute}\left(t\right)-k_{out,acute}\cdot Response\left(t\right),$$

(5) $$E_{L-OHP,acute}\left(t\right)=\frac{E_{\max,acute}\cdot C^{\gamma acute}\left(t\right)}{EC_{50,acute}{}^{\gamma acute}+C^{\gamma acute}\left(t\right)},$$

where *k_in,acute_* was a zero-order rate constant describing the rate of increase of paw withdrawal responses, *k_out, acute_* was a first-order rate constant describing the rate of decrease of paw withdrawal responses, *E_L-OHP,acute_* was the drug effect for acute neuropathy, *E_max,acute_* was the maximum drug effect for acute neuropathy, and *EC*_50,*acute*_ was the L-OHP concentration when the drug effect was at half of *E_max,acute_*. In our preliminary studies, the value *γ_acute_* = 6 allowed the TD model to effectively capture the features of TD data. To minimize the estimated number of parameters, the value of *γ_acute_* was fixed at 6. The TD model development process for acute neuropathy is presented in Table S1 and Figure S2.

#### 2.2.4. TD Model for Chronic Neuropathy

Based on the reported mechanisms of L-OHP-induced chronic neuropathy, the values of paw withdrawal threshold after exposure to von Frey filaments were fitted with a transit compartment model [36,37]. This model can describe delay of neuronal cell injury and onset of chronic neuropathy with respect to L-OHP treatment. It was assumed that neuronal cell exposure to L-OHP passed through *n* different stages (named *x*_1_, …, *x_n_*), characterized by progressive degrees of damage, resulting in chronic neuropathy. The developed TD model assumed that L-OHP decreased paw withdrawal threshold by atrophy of neurons. This feature was described by the following equation:

(6) $$\frac{dx_{1}\left(t\right)}{dt}=k_{in,\;chronic}\cdot \left(1-E_{L-OHP,chronic}\left(t\right)\right)-k_{out,chronic}\cdot x_{1}\left(t\right),\;x_{chronic}\left(0\right)=x_{0,chronic},$$

(7) $$\frac{dx_{2}\left(t\right)}{dt}=k_{out,\;chronic}\cdot x_{1}\left(t\right)-k_{out,\;chronic}\cdot x_{2}\left(t\right),$$

(8) $$\frac{dx_{n}\left(t\right)}{dt}=k_{out,\;chronic}\cdot x_{n-1}\left(t\right)-k_{out,\;chronic}\cdot x_{n}\left(t\right),\;n\;=\;3,\;4,\;5,$$

(9) $$\frac{dx_{chronic}\left(t\right)}{dt}=k_{out,\;chronic}\cdot x_{5}\left(t\right)-k_{out,\;chronic}\cdot x_{chronic}\left(t\right),$$

(10) $$k_{out,chronic}=k_{in,chronic}/x_{0},$$

(11) $$E_{L-OHP,chronic}\left(t\right)=\frac{E_{\max,chronic}\cdot C^{\gamma chronic}\left(t\right)}{EC_{50,chronic}{}^{\gamma chronic}+C^{\gamma chronic}\left(t\right)},$$

where *x_chronic_*(*t*) was the paw withdrawal threshold, *k_in,chronic_* was a zero-order rate constant describing the rate of increase of paw withdrawal threshold, *k_out,chronic_* was a first-order rate constant describing the rate of decrease of paw withdrawal threshold, *x*_0,*chronic*_ was the baseline value of the responses, *E_L-OHP,chronic_* was the drug effect on chronic neuropathy, *E_max,chronic_* was the maximum drug effect for chronic neuropathy, and *EC*_50,*chronic*_ was the L-OHP concentration when the drug effect was at half of *E_max,chronic_*. Based on the observed data in the control group, the baseline value of the responses was 8 g. In our preliminary studies, the values *n* = 5 and *γ_chronic_* = 4 allowed the TD model to effectively capture TD. To minimize the estimated number of parameters, the value of *x*_0,*chronic*_, *n*, and *γ_chronic_* was fixed at 8, 5, and 4, respectively. The TD model development process for chronic neuropathy is presented in Table S2 and Figure S3.

Similar to PK modeling, different inter-individual variability models of TD parameters and residual variability models in the TD data were initially tested and assumed using exponential and proportional error model, respectively. The diagnosis plots of TD model are shown in Figure S4.

### 2.3. Model Evaluation

A prediction-corrected visual predictive check [38] and a nonparametric bootstrap procedure were performed to check the stability of the final PK-TD model using Phoenix^®^ NLME™ Version 8.1 software (Certara USA, Inc., Princeton, NJ, USA). In the visual predictive check, data sets (*n* = 1000) were simulated using the final population model parameters, and the 5th, 50th, and 95th simulated percentiles of L-OHP concentration, and quantitative TD data, were calculated. For the nonparametric bootstrap procedure, data sets (*n* = 1000) were simulated and compared with the population model parameters estimated from the original data set.

### 2.4. Simulation to Assess the Effects of Dosing Schedule on Neuropathy

To determine the effect of dosing schedule on time-course profiles of degree of acute and chronic neuropathy, we plotted simulated curves relative to different dosing schedules. Simulations were performed using the final PK–TD model and final fixed effect parameters (Table 1 and Table 2). The number of paw withdrawal responses in the acetone test and the paw withdrawal threshold in the von Frey filament test were simulated after administration of placebo or L-OHP (1–8 mg/kg) once per week for four weeks, L-OHP (5 mg/kg) every week (Day 0, 7, 14, and 21) at two week intervals (Day 0 and 14), or at three week intervals (Day 0 and 21).

## 3. Results

### 3.1. PK Model

**Figure 2:**
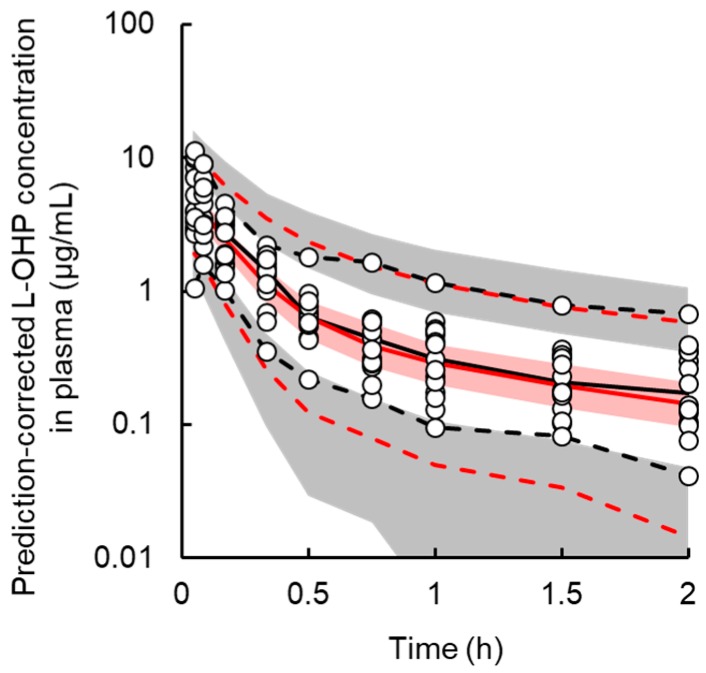
Prediction-corrected visual predictive check plot for the final pharmacokinetic model of oxaliplatin (L-OHP) in rats. The solid black and red line represents the median observed and simulated prediction-corrected plasma concentration, respectively, and the semitransparent red field represents a simulation-based 95% confidence interval for the median. The observed and simulated 5% and 95% percentiles are presented with dashed black and red lines, respectively, and the 95% confidence intervals for the corresponding model predicted percentiles are shown as semitransparent gray fields. The observed prediction-corrected plasma concentrations are represented by circles.

A two-compartment model with linear elimination best described the PK of L-OHP. The final PK parameter estimates and results of the bootstrap validation are shown in Table 1 and Figure S5. The coefficient of variation (CV%) of each PK parameter estimate was ≤34.2%. The CV% for the inter-individual variability parameter was large (≤148.5%). This result was expected because the number of animals (*n* = 15) in the PK study was small and precise estimation of inter-individual variability parameters was difficult. This is a minor concern since the stability of the PK model was validated by the bootstrap procedure. Each parameter estimate obtained from the bootstrap validation was similar to those from the original data set, suggesting that the PK model adequately estimated the parameters. Figure 2 presents the results of prediction-corrected visual predictive evaluation of the final PK model. Although there was a relatively large inter-individual variability in the low-dose group, visual predictive check plots indicated that the PK model was sufficient to describe individual plasma L-OHP concentrations.

### 3.2. TD Model for Acute Neuropathy

**Figure 3:**
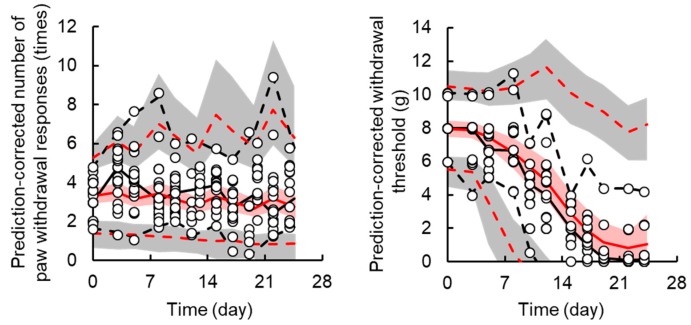
Prediction-corrected visual predictive check plot for the final pharmacokinetic-toxicodynamic (PK-TD) model of oxaliplatin (L-OHP)-induced acute (the **left panel**) and chronic (the **right panel**) neuropathy in rats. The solid black and red line represents the median observed and simulated prediction-corrected plasma concentration, respectively, and the semitransparent red field represents a simulation-based 95% confidence interval for the median. The observed and simulated 5% and 95% percentiles are presented with dashed black and red lines, respectively, and the 95% confidence intervals for the corresponding model predicted percentiles are shown as semitransparent gray fields. The observed prediction-corrected number of paw withdrawal responses in the acetone test and paw withdrawal threshold in the von Frey filament test are represented by circles.

**Figure 4:**
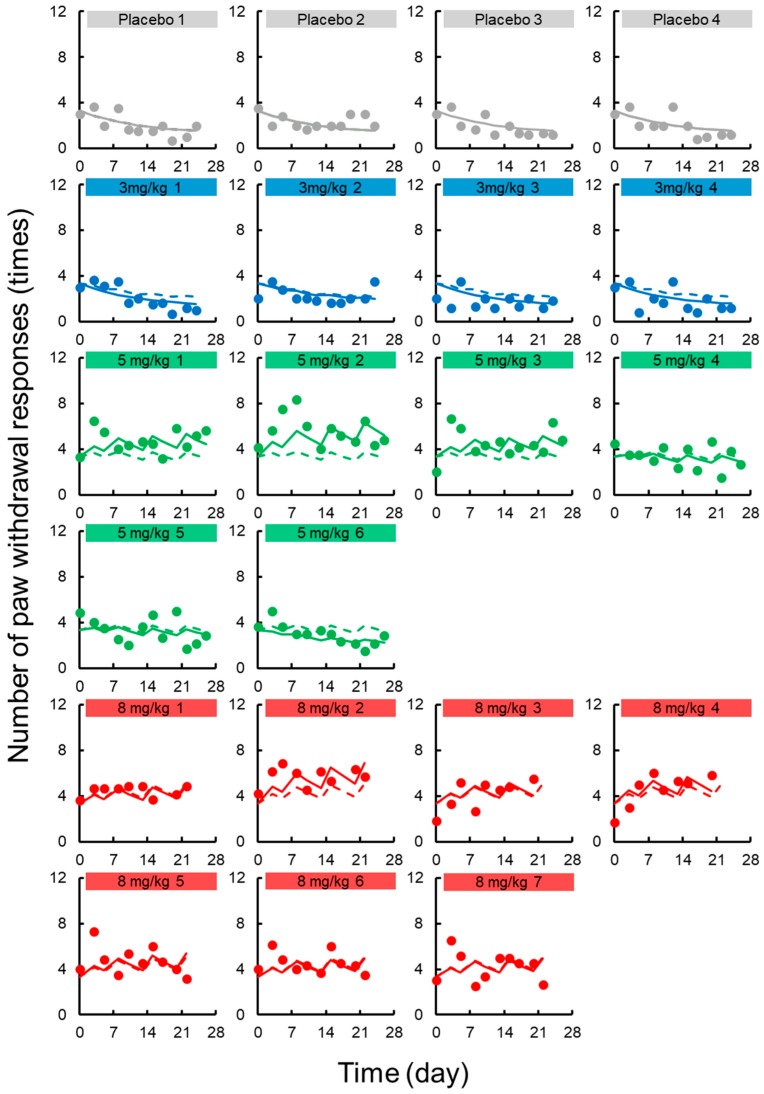
Individual number of paw withdrawal responses in the acetone test vs. time profiles after intravenous administration of oxaliplatin (L-OHP) to rats. Each circle represents the observed number of paw withdrawal responses in the acetone, with a fitted solid line depicting the individual number of paw withdrawal responses predicted by the PK-TD model and a dashed line showing the population fits.

The TD model with a sigmoid-*E_max_* model successfully described the handling and drug effects on the number of paw withdrawal responses in the acetone test. Random effects of TD parameters could not be explained by the model due to high η-shrinkage values. Based on the model development criteria, random effects were not applied to the TD model of acute neuropathy. Figure 3 shows the results of the prediction-corrected visual predictive check for the final PK-TD model for acute neuropathy. The models accurately predicted paw withdrawal threshold in the acetone test as most observations are within the 95% prediction interval. Figure 4 shows individual and population prediction of number of paw withdrawal responses in the acetone test vs. time profiles after intravenous administration of L-OHP to rats. Although the PK-TD model underestimated the increase in number of paw withdrawal responses in the acetone test at the initial phase (≤Day 7) after administration of high dose of L-OHP, the model well described individual TD data. The final TD parameter estimates for acute neuropathy and the results of the bootstrap validation are summarized in Table 2 and Figure S5. The CV% of each fixed effect parameter estimate and the residual variability parameter were ≤47.2%, suggesting that these parameters were estimated well. The results of the predictive check and bootstrap validation indicated that the model adequately predicted the individual number of paw withdrawal responses in the acetone test.

### 3.3. TD Model for Chronic Neuropath

**Figure 5:**
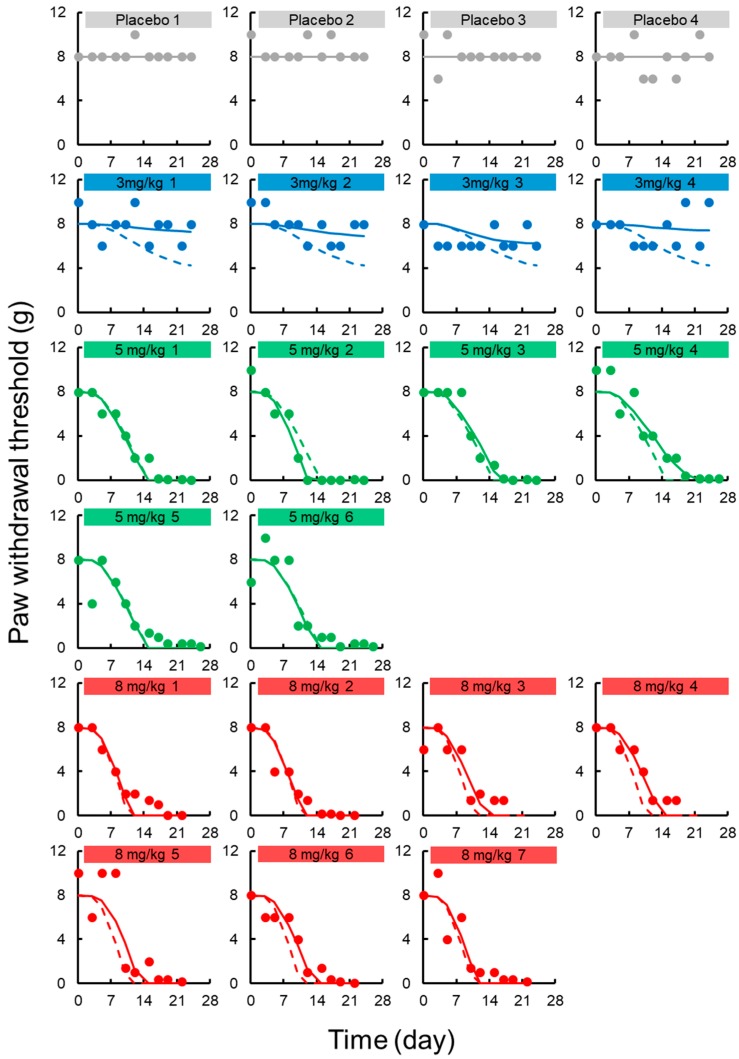
Individual paw withdrawal threshold in the von Frey filament test vs. time profiles after intravenous administration of oxaliplatin (L-OHP) to rats. Each circle represents the observed paw withdrawal threshold in the von Frey filament test, with a fitted solid line depicting the individual paw withdrawal threshold predicted by the PK-TD model and a dashed line showing the population fits.

A transit compartment model was applied for describing the delay in onset of chronic neuropathy. Random effects were applied for *k_in,chronic_* and *EC*_50,*chronic*_, and successfully contributed to prediction of individual decreases in paw withdrawal threshold in the von Frey test. Figure 3 shows the results of prediction-corrected visual predictive assessment of the final PK-TD model. Figure 5 shows individual and population prediction of paw withdrawal threshold in the von Frey filament test vs. time profiles after intravenous administration of L-OHP to rats. Although there were instances of overestimation and underestimation in the prediction-corrected withdrawal threshold in the results of predictive check, the models well predicted paw withdrawal threshold in the von Frey test, as most observations are within the 95% prediction interval. The model adequately described individual TD data. The CV% of each fixed effect parameter estimate and inter-individual variability were ≤0.67% and ≤40.5, respectively. Each parameter estimate obtained by the bootstrap procedure was similar to those obtained by the original data set, suggesting that these parameters were estimated with good precision.

### 3.4. Simulation to Assess the Effects of Dosing Schedule on Neuropathy

**Figure 6:**
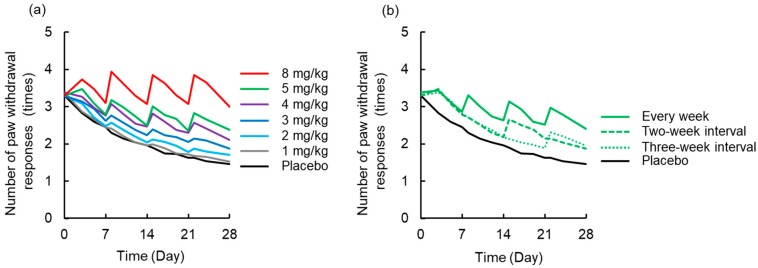
Simulated time-course profile obtained using the pharmacokinetic-toxicodynamic (PK-TD) model for the number of paw withdrawal responses in the acetone test. (**a**) Simulated data after administration of placebo or L-OHP (1–8 mg/kg) once per week for four weeks, (**b**) L-OHP (5 mg/kg) every week, with two-week interval (Day 0 and 14), and three-week interval (Day 0 and 21). PK-TD parameters: *V* = 357.8 mL·kg^−1^, *k_e_* = 3.4 h^−1^, *k*_12_ = 2.6 h^−1^, *k*_21_ = 1.1 h^−1^, *k_in,acute_* = 0.004 day^−1^, *k_out,acute_* = 0.06 day^−1^, *k_handling_* = 0.08 day^−1^, *E_max,acute_* = 10.1, *EC*_50,*acute*_ = 0.36 μg·mL^−1^, *γ_acute_* = 6.

**Figure 7:**
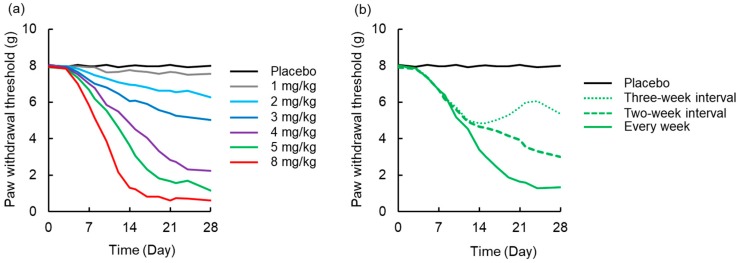
Simulated time-course profile obtained using the pharmacokinetic-toxicodynamic (PK-TD) model for paw withdrawal threshold in the von Frey filament test. (**a**) Simulated data after administration of placebo or L-OHP (1-8 mg/kg) once per week for four weeks, (**b**) L-OHP (5 mg/kg) every week, at two-week interval (Day 0 and 14), and three-week interval (Day 0 and 21). PK-TD parameters: *V* = 357.8 mL·kg^−1^, *k_e_* = 3.4 h^−1^, *k*_12_ = 2.6 h^−1^, *k*_21_ = 1.1 h^−1^, *k_in,chronic_* = 2.7 day^−1^, *E_max,chronic_* = 168.0, *EC*_50,*chronic*_ = 0.43 μg·mL^−1^, *γ_chronic_* = 4.

A simulated time-course profile obtained using the PK-TD model for number of paw withdrawal responses in the acetone test and paw withdrawal threshold in the von Frey test after different doses of L-OHP and different washout periods are shown in Figure 6 and Figure 7. According to the simulation results for L-OHP-induced acute neuropathy, cold allodynia was predicted to occur more intensively with increased dose, but onset time was not altered. However, the degree of cold allodynia mildly decreased with a longer washout period. Similar to simulation of acute neuropathy, mechanical allodynia was exacerbated by increased dose and was mitigated by a longer washout period. The onset of mechanical allodynia was not dependent on the L-OHP dose.

## 4. Discussion

In the current study, we successfully developed, for the first time, a PK-TD model of L-OHP for peripheral neuropathy in rats. The population disposition of L-OHP in rats was well described by a two-compartment model with linear elimination, which is in accordance with a previous report [31]. In a population PK model analysis of L-OHP in patients, three or more compartment models were selected for describing the time-course of plasma concentration of L-OHP [39,40]. These results indicated that selection of an appropriate PK model for L-OHP would be needed when extending the range of applications of the current PK-TD model to clinical data analysis.

Based on different mechanisms of onset of L-OHP-induced peripheral neuropathy, a TD model was developed independently. Acute neuropathy is induced by alterations in voltage-gated Na^+^ and K^+^ channels [11,13], and involvement of transient receptor potential-Ankyrin 1 and -melastatin 8 [41] but not by neuronal injury. In contrast, chronic neuropathy is caused by primary sensory neuron neurotoxicity [42]. However, the detailed mechanisms of development of acute or chronic L-OHP-induced neuropathy are unknown. Although a limitation is the difficulty in applying results of the preclinical model directly to predicting the toxicities in patients, the evaluation of drug-induced neuropathy using the nociception animal models is valuable in order to provide meaningful results for understanding certain mechanisms involved in this response. In the current study, we constructed a PK-TD model employing a small number of typical parameters. This study may provide an experimental clue to understanding the mechanisms of L-OHP-induced peripheral neuropathy.

A model with a sigmoid-*E_max_* model best described the results of the acetone test and successfully linked the L-OHP dosing regimen to cold allodynia. The acetone test has been widely used to characterize the mechanisms of L-OHP-induced cold allodynia in animal models, and to identify therapeutic agents for use to treat cold allodynia following various L-OHP dosing regimens [43]. The current model might allow for greater understanding of the relationship between dosing regimen and cold allodynia. PK-TD model simulation results suggested that increased dosing worsened acute neuropathy and longer washout times aid in recovery from neurotoxicity, which is consistent with clinical observations. Moderate to severe acute L-OHP-induced neuropathy symptoms (≥grade 2) were common in patients who were given large starting doses of L-OHP (>85 mg/m^2^) [44,45]. However, no study has examined the range (or minimal) dose required to evoke acute neuropathy [46]. A prospective study using PK-TD modeling and simulation might provide valuable insights to aid in determination of more appropriate initial dosing and/or infusion times to prevent or alleviate symptoms.

For the TD model of chronic neuropathy, a transit compartment model was applied and accurately described alteration of paw withdrawal threshold in the von Frey test. Transit compartment models are widely used for modeling pharmacological effects of compounds that may be mediated by time-dependent transduction, and when there is a time lag in the final drug response [47]. In TD studies of anticancer agents, the transit compartment model is valuable for describing the process of tumor cell death and characterizing myelosuppression [48,49]. Carozzi et al. have reported that the oxidative stress induces neuronal and glial cell dysfunction and cell death induced by caspases, mitogen activated protein kinases and protein kinase C; these transit processes may be mechanisms for L-OHP-induced chronic neuropathy [50]. Our results support this hypothesis for mechanisms of L-OHP-induced chronic neuropathy. The results of the current study suggested that the transit compartment model could be expanded to characterize L-OHP-induced chronic neuropathy and to analyze mechanical allodynia data. The irreversible effect model [51] is generally used to describe cell killing actions of chemotherapeutic agents, and the model was also a candidate for the TD model for chronic neuropathy developed in this study because chronic neuropathy can be irreversible [8]. However, the irreversible effect model did not sufficiently describe our observed TD data of chronic neuropathy, possibly due to a short observation period. To improve the TD model, further studies with long-term observation periods after the final L-OHP administration might useful.

In preclinical TD studies of anticancer agents, large inter-individual variability of TD parameters was reported. In a PK-TD model used to characterize the disposition of topotecan and its toxicity, inter-individual variability in body weight loss was 48.6–188% [34]. Inter-individual variability in TD model parameters of 5-fluorouracil (5-FU) (≤82.6%) was higher than that in PK model parameters (≤47.5%), suggesting that individual fluctuations in cell sensitivity to 5-FU could affect onset and degree of drug-induced toxicity [32]. However, in the current study, inter-individual variability in drug effects in the TD model for acute and chronic neuropathy was undetermined and ≤28.2%, respectively, which was unexpectedly lower than values obtained using the PK model. These results suggested that individual fluctuations in L-OHP concentration in plasma might be a more important factor for individual variability of onset and degree of neuropathy than that of cell sensitivity to L-OHP.

The “stop-and-go” strategy is a valuable approach for achieving low incidence of severe neurotoxicity while maintaining treatment efficacy [52]. However, determination of an appropriate dose and period of stopping L-OHP administration is a critical challenge. In the current study, we successfully developed a PK-TD model that quantitatively simulates the relationship between washout period following L-OHP dosing and onset of peripheral neuropathy. Therefore, the current model would be valuable for investigation and simulation of dosing strategies, including washout period after L-OHP administration for individual patients. Based on our results using our PK-TD model, we proposed that a long washout period is a good strategy to avoid onset of severe mechanical allodynia. However, to achieve individual chemotherapeutic strategies that reduce neuropathy without disease progression, clinical studies including toxicodynamic evaluation with a large sample size are needed.

This is the first report of a PK-TD model to characterize the relationship between the disposition of L-OHP and drug-induced neuropathy. The presented results can aid in development of optimal dosing regimens for treatment of cancer. However, this study had several limitations. First, the current model partially under- or over-estimated the individual observed TD data, possibly due to small sample size. In the current study, total platinum concentrations in plasma were used to develop the PK-TD model. However, L-OHP is rapidly transformed into a dichloro-1,2-diaminocyclohexylplatinum complex and oxalate [53], and accumulation of L-OHP in plasma has been observed after multiple doses of L-OHP to rats [23]. Although the current PK-TD model was a simple model, application of fluctuating factors to the PK-TD model might improve estimation of individual TD data. Second, the source data for our study was obtained in healthy rats, not gastric, colorectal, or pancreatic cancer model rats. There is a possibility that cancer conditions affect PK and TD of L-OHP. To further characterize the relationship between PK and L-OHP-induced neuropathy, modeling using data from rat cancer models is needed. Finally, it is difficult to apply our results directly to analysis of practical data in patients since grade of neuropathy in patients is assessed using an ordinal scale (i.e., Grade 1–4 by Common Terminology Criteria for Adverse Events), not a continuous scale. The next step towards developing an optimal dosing strategy is population PK-TD model analysis using patients’ data based on the current results and investigations of the effects of washout period on the degree of the L-OHP-induced neuropathy in patients. Development of PK-TD model that can assess grade of toxicity in patients remains an issue for further study. To construct the optimal dosing strategy for L-OHP-based chemotherapy, clinical trials with large sample sizes and further PK-TD model development based on the current results are required.

## 5. Conclusions

We successfully developed a PK-TD model for prediction of L-OHP-induced acute and chronic neuropathy and determined the quantitative relationship between dosing regimen and magnitude of neuropathy. Our results provide a framework for future investigations of optimal dosing strategies that can reduce neuropathy while maintaining the therapeutic effects of L-OHP chemotherapy. Moreover, the current model might contribute the assessments of neuropathy data in combined applications of preventative agents or novel anticancer agents. Thus, our findings might have important implications for individualized dosing regimens of anticancer agents. However, to construct an optimal L-OHP dosing regimen, further preclinical and clinical studies are needed.

## Figures and Tables

**Table 1:** PK parameters of L-OHP in rats after administration of 3, 5, or 8 mg/kg L-OHP.

Parameters	Unit	Final Model		Bootstrap			
		Estimate	CV%	Median	5th–95th Percentiles		
Fixed effect parameters, θ							
*V*	mL·kg^−1^	357.8	15.7	354.8	284.2	-	410.9
*ke*	h^−1^	3.4	15.1	3.4	2.5	-	4.2
*k*_12_	h^−1^	2.6	34.2	2.7	1.9	-	4.3
*k*_21_	h^−1^	1.1	30.1	1.1	0.6	-	1.5
Inter-individual variability, ω							
*V*	%	42.4	47.0	41.5	24.1	-	50.4
*ke*	%	40.6	93.9	36.4	0.01	-	57.0
*k*_12_	%	54.3	148.5	50.2	0.01	-	95.2
*k*_21_	%	91.0	56.8	91.0	58.5	-	130.8
Residual variability, σ							
*C*	%	21.9	13.1	22.0	17.9	-	25.7

*V*, central volume of the distribution; *k_e_*, elimination rate constant from the central compartment; *k*_12_, rate constant of the central compartment to the peripheral compartment; *k*_21_, rate constant of the peripheral compartment to the central compartment; *C*, concentration of L-OHP in the central compartment. The value of ω is expressed as percent coefficient of variation.

**Table 2:** PD parameters of acute and chronic neuropathy after administration of L-OHP in rats.

Parameters	Unit	Final Model		Bootstrap			
		Estimate	CV%	Median	2.5th–97.5th Percentiles		
*Acute neuropathy*							
Fixed effect parameters, θ							
*x*_0,*acute*_	times	3.1	Fix	3.1	Fix		
*k_in,acute_*	times·day^−1^	0.004	47.2	0.004	0.0001	-	0.019
*k_out,acute_*	day^−1^	0.06	24.2	0.06	0.03	-	0.09
*k_handling_*	day^−1^	0.08	Fix	0.08	Fix		
*E* *_max,acute_*	times·day^−1^	10.1	20.5	9.9	5.3	-	14.0
*EC*_50*,acute*_	μg·mL^−1^	0.36	11.3	0.35	0.23	-	0.40
*γ_acute_*		6	Fix	6	Fix		
Residual variability, σ							
*x_acute_* in Proportional error	%	32.1	3.5	31.9	29.9	-	33.9
*Chronic neuropathy*							
Fixed effect parameters, θ							
*x*_0,*chronic*_	g	8	Fix	8	Fix		
*k_in,chronic_*	g·day^−1^	2.7	0.66	2.8	2.4	-	3.7
*E* *_max,chronic_*		168.0	0.67	127.7	29.8	-	169.3
*EC*_50,*chronic*_	μg·mL^−1^	0.43	0.67	0.35	0.21	-	0.68
*γ_chronic_*		4	Fix	4	Fix		
Inter-individual variability, ω							
*k_in,chronic_*	%	25.2	27.8	25.1	4.4	-	25.5
*EC*_50,*chronic*_	%	28.2	40.5	27.9	0.001	-	28.2
Residual variability, σ							
*x_chronic_* in Additive error	g	1.3	5.9	1.3	1.1	-	1.4

*x*_0,*acute*_, the baseline value of paw withdrawal responses in acetone test; *k_in,acute_*, a zero-order rate constant describing the rate of paw withdrawal responses increase; *k_out,acute_*, a first-order rate constant describing the rate of decrease of paw withdrawal responses; *k_handling_*, constant in handling effect; *E_max,acute_*, the maximum drug effect for acute neuropathy; *EC*_50,*acute*_, the L-OHP concentration when the drug effect was at half of *E_max,acute_*; *γ_acute_*, hill coefficient for acute neuropathy model; *x*_0,*chronic*_, the baseline value of paw withdrawal threshold in von Frey test; *k_in,chronic_*, a zero-order rate constant describing the rate of paw withdrawal threshold increase; *E_max,chronic_*, the maximum drug effect for chronic neuropathy; *EC*_50*,chronic*_, the L-OHP concentration when the drug effect was at half of *E_max,chronic_*; *γ_chronic_*, hill coefficient for chronic neuropathy model. The value of ω is expressed as percent coefficient of variation.

## Supplementary Materials

The following are available online at https://www.mdpi.com/1999-4923/12/2/125/s1, Figure S1. Goodness-of-fit plots of final population pharmacokinetic model of oxaliplatin (L-OHP). Population predictions (PRED) vs. observations (OBS), individual predictions (IPRED) vs. OBS, conditional weighed residuals (CWRES) vs. time, and CWRES vs. IPRED. Figure S2. Fitted results of preliminary developed toxicodynamic (TD) model of oxaliplatin (L-OHP) for acute neuropathy. Figure S3. Fitted results of preliminary developed toxicodynamic (TD) model of oxaliplatin (L-OHP) for chronic neuropathy. Figure S4. Goodness-of-fit plots of final population pharmacokinetic-toxicodynamic model of oxaliplatin (L-OHP). Population predictions (PRED) vs. observations (OBS), individual predictions (IPRED) vs. OBS, conditional weighed residuals (CWRES) vs. time, and CWRES vs. IPRED. Figure S5. Distribution of final pharmacokinetic-toxicodynamic (PK-TD) model parameters obtained by Bootstrap procedure (*n* = 1000). Table S1: Toxicodynamic model selection for acute neuropathy. Table S2. Toxicodynamic model selection for chronic neuropathy.

## References

[1] TAndré T., Boni C., Mounedji-Boudiaf L., Navarro M., Tabernero J., Hickish T., Topham C., Zaninelli M., Clingan P., Bridgewater J. (2004). Multicenter International Study of Oxaliplatin/5-Fluorouracil/Leucovorin in the Adjuvant Treatment of Colon Cancer (MOSAIC) Investigators. Oxaliplatin, fluorouracil, and leucovorin as adjuvant treatment for colon cancer. N. Engl. J. Med..

[2] Pinter T., Klippel Z., Cesas A., Croitoru A., Decaestecker J., Gibbs P., Hotko Y., Jassem J., Kurteva G., Novotny J. (2017). Phase III, Randomized, Double-Blind, Placebo-Controlled Trial of Pegfilgrastim in Patients Receiving First-Line FOLFOX/Bevacizumab or FOLFIRI/Bevacizumab for Locally Advanced or Metastatic Colorectal Cancer: Final Results of the Pegfilgrastim and Anti-VEGF Evaluation Study (PAVES). Clin. Colorectal Cancer.

[3] Degirmencioglu S., Tanrıverdi O., Demiray A.G., Senol H., Dogu G.G., Yaren A. (2019). Retrospective comparison of efficacy and safety of CAPOX and FOLFOX regimens as adjuvant treatment in patients with stage III colon cancer. J. Int. Med. Res..

[4] Chen L., Hao Y., Cong X., Zou M., Li S., Zhu L., Song H., Xue Y. (2019). Peripheral Venous Blood Platelet-to-Lymphocyte Ratio (PLR) for Predicting the Survival of Patients with Gastric Cancer Treated with SOX or XELOX Regimen Neoadjuvant Chemotherapy. Technol. Cancer Res. Treat..

[5] Ahmed F., Kumari S., Kondapi A.K. (2018). Evaluation of Antiproliferative Activity, Safety and Biodistribution of Oxaliplatin and 5-Fluorouracil Loaded Lactoferrin Nanoparticles for the Management of Colon Adenocarcinoma: An In Vitro and an In Vivo Study. Pharm. Res..

[6] Hanada K., Suda M., Kanai N., Ogata H. (2010). Pharmacokinetics and toxicodynamics of oxaliplatin in rats: Application of a toxicity factor to explain differences in the nephrotoxicity and myelosuppression induced by oxaliplatin and the other platinum antitumor derivatives. Pharm. Res..

[7] McWhinney S.R., Goldberg R.M., McLeod H.L. (2009). Platinum neurotoxicity pharmacogenetics. Mol. Cancer Ther..

[8] Avan A., Postma T.J., Ceresa C., Avan A., Cavaletti G., Giovannetti E., Peters G.J. (2015). Platinum-induced neurotoxicity and preventive strategies: Past, present, and future. Oncologist.

[9] Nakagawa T., Kaneko S. (2017). Roles of Transient Receptor Potential Ankyrin 1 in Oxaliplatin-Induced Peripheral Neuropathy. Biol. Pharm. Bull..

[10] Adelsberger H., Quasthoff S., Grosskreutz J., Lepier A., Eckel F., Lersch C. (2000). The chemotherapeutic oxaliplatin alters voltage-gated Na(+) channel kinetics on rat sensory neurons. Eur. J. Pharmacol..

[11] Benoit E., Brienza S., Dubois J.M. (2006). Oxaliplatin, an anticancer agent that affects both Na+ and K+ channels in frog peripheral myelinated axons. Gen. Physiol. Biophys..

[12] Grolleau F., Gamelin L., Boisdron-Celle M., Lapied B., Pelhate M., Gamelin E. (2001). A possible explanation for a neurotoxic effect of the anticancer agent oxaliplatin on neuronal voltage-gated sodium channels. J. Neurophysiol..

[13] Kagiava A., Tsingotjidou A., Emmanouilides C., Theophilidis G. (2008). The effects of oxaliplatin, an anticancer drug, on potassium channels of the peripheral myelinated nerve fibres of the adult rat. Neurotoxicology.

[14] Cersosimo R.J. (2005). Oxaliplatin-associated neuropathy: A review. Ann. Pharmacother..

[15] Argyriou A.A., Cavaletti G., Antonacopoulou A., Genazzani A.A., Briani C., Bruna J., Terrazzino S., Velasco R., Alberti P., Campagnolo M. (2013). Voltage-gated sodium channel polymorphisms play a pivotal role in the development of oxaliplatin-induced peripheral neurotoxicity: Results from a prospective multicenter study. Cancer.

[16] Grothey A., Nikcevich D.A., Sloan J.A., Kugler J.W., Silberstein P.T., Dentchev T., Wender D.B., Novotny P.J., Chitaley U., Alberts S.R. (2011). Intravenous calcium and magnesium for oxaliplatin-induced sensory neurotoxicity in adjuvant colon cancer: NCCTG N04C7. J. Clin. Oncol..

[17] Kurniali P.C., Luo L.G., Weitberg A.B. (2010). Role of calcium/magnesium infusion in oxaliplatin-based chemotherapy for colorectal cancer patients. Oncology.

[18] Dworkin R.H., O’Connor A.B., Backonja M., Farrar J.T., Finnerup N.B., Jensen T.S., Kalso E.A., Loeser J.D., Miaskowski C., Nurmikko T.J. (2007). Pharmacologic management of neuropathic pain: Evidence-based recommendations. Pain.

[19] Rao R.D., Michalak J.C., Sloan J.A., Loprinzi C.L., Soori G.S., Nikcevich D.A., Warner D.O., Novotny P., Kutteh L.A., Wong G.Y. (2007). North Central Cancer Treatment Group. Efficacy of gabapentin in the management of chemotherapy-induced peripheral neuropathy: A phase 3 randomized, double-blind, placebo-controlled, crossover trial (N00C3). Cancer.

[20] Kanat O., Ertas H., Caner B. (2017). Platinum-induced neurotoxicity: A review of possible mechanisms. World J Clin. Oncol..

[21] Saif M.W., Reardon J. (2005). Management of oxaliplatin-induced peripheral neuropathy. Ther. Clin. Risk Manag..

[22] Jamieson S.M., Liu J., Connor B., McKeage M.J. (2005). Oxaliplatin causes selective atrophy of a subpopulation of dorsal root ganglion neurons without inducing cell loss. Cancer Chemother. Pharmacol..

[23] Ito Y., Kobuchi S., Shimizu R., Katsuyama Y. (2018). Pharmacokinetic and toxicodynamic evaluation of oxaliplatin-induced neuropathy and hematological toxicity in rats. Cancer Chemother. Pharmacol..

[24] Kobuchi S., Katsuyama Y., Ito Y. (2019). Mechanism-based pharmacokinetic-pharmacodynamic (PK-PD) modeling and simulation of oxaliplatin for hematological toxicity in rats. Xenobiotica.

[25] Kobuchi S., Ito Y., Hayakawa T., Nishimura A., Shibata N., Takada K., Sakaeda T. (2014). Pharmacokinetic-pharmacodynamic (PK-PD) modeling and simulation of 5-fluorouracil for erythropenia in rats. J. Pharmacol. Toxicol. Methods.

[26] Friberg L.E., Freijs A., Sandström M., Karlsson M.O. (2000). Semiphysiological model for the time course of leukocytes after varying schedules of 5-fluorouracil in rats. J. Pharmacol. Exp. Ther..

[27] Fujita S., Ushio S., Ozawa N., Masuguchi K., Kawashiri T., Oishi R., Egashira N. (2015). Exenatide Facilitates Recovery from Oxaliplatin-Induced Peripheral Neuropathy in Rats. PLoS ONE.

[28] Minakata K., Nozawa H., Suzuki M., Gonmori K., Yamagishi I., Watanabe K., Suzuki O. (2007). Trace analysis of platinum in blood and urine by ESI-MS-MS. Forensic Toxicol..

[29] Flatters S.J., Bennett G.J. (2004). Ethosuximide reverses paclitaxeland vincristine-induced painful peripheral neuropathy. Pain.

[30] Di Cesare Mannelli L., Pacini A., Bonaccini L., Zanardelli M., Mello T., Ghelardini C. (2013). Morphologic features and glial activation in rat oxaliplatin-dependent neuropathic pain. J. Pain.

[31] Mould D.R., Upton R.N. (2013). Basic concepts in population modeling, simulation, and model-based drug development-part 2: Introduction to pharmacokinetic modeling methods. CPT Pharmacometri. Syst. Pharmacol..

[32] Mas-Fuster M.I., Ramon-Lopez A., Lacueva F.J., Arroyo A., Más-Serrano P., Nalda-Molina R. (2018). Population pharmacokinetics of oxaliplatin after intraperitoneal administration with hyperthermia in Wistar rats. Eur. J. Pharm. Sci..

[33] Kobuchi S., Ito Y., Sakaeda T. (2017). Population Pharmacokinetic-Pharmacodynamic Modeling of 5-Fluorouracil for Toxicities in Rats. Eur. J. Drug Metab. Pharmacokinet..

[34] Chen J., Lu Q., Balthasar J.P. (2007). Mathematical modeling of topotecan pharmacokinetics and toxicodynamics in mice. J. Pharmacokinet. Pharmacodyn..

[35] Gao W., Jusko W.J. (2012). Modeling disease progression and rosiglitazone intervention in type 2 diabetic Goto-Kakizaki rats. J. Pharmacol. Exp. Ther..

[36] Sharma A., Jusko W.J. (1998). Characteristics of indirect pharmacodynamic models and applications to clinical drug responses. Br. J. Clin. Pharmacol..

[37] Mager D.E., Jusko W.J. (2001). Pharmacodynamic modeling of time-dependent transduction systems. Clin. Pharmacol. Ther..

[38] Bergstrand M., Hooker A.C., Wallin J.E., Karlsson M.O. (2011). Prediction-corrected visual predictive checks for diagnosing nonlinear mixed-effects models. AAPS J..

[39] Chalret du Rieu Q., White-Koning M., Picaud L., Lochon I., Marsili S., Gladieff L., Chatelut E., Ferron G. (2014). Population pharmacokinetics of peritoneal, plasma ultrafiltrated and protein-bound oxaliplatin concentrations in patients with disseminated peritoneal cancer after intraperitoneal hyperthermic chemoperfusion of oxaliplatin following cytoreductive surgery: Correlation between oxaliplatin exposure and thrombocytopenia. Cancer Chemother. Pharmacol..

[40] Nikanjam M., Stewart C.F., Takimoto C.H., Synold T.W., Beaty O., Fouladi M., Capparelli E.V. (2015). Population pharmacokinetic analysis of oxaliplatin in adults and children identifies important covariates for dosing. Cancer Chemother. Pharmacol..

[41] Gauchan P., Andoh T., Kato A., Kuraishi Y. (2009). Involvement of increased expression of transient receptor potential melastatin 8 in oxaliplatin-induced cold allodynia in mice. Neurosci. Lett..

[42] Zhao M., Nakamura S., Miyake T., So K., Shirakawa H., Tokuyama S., Narita M., Nakagawa T., Kaneko S. (2014). Pharmacological characterization of standard analgesics on oxaliplatin-induced acute cold hypersensitivity in mice. J. Pharmacol. Sci..

[43] Ushio S., Egashira N., Sada H., Kawashiri T., Shirahama M., Masuguchi K., Oishi R. (2012). Goshajinkigan reduces oxaliplatin-induced peripheral neuropathy without affecting anti-tumour efficacy in rodents. Eur. J. Cancer.

[44] Storey D.J., Sakala M., McLean C.M., Phillips H.A., Dawson L.K., Wall L.R., Fallon M.T., Clive S. (2010). Capecitabine combined with oxaliplatin (CapOx) in clinical practice: How significant is peripheral neuropathy?. Ann. Oncol..

[45] Schmoll H.J., Cartwright T., Tabernero J., Nowacki M.P., Figer A., Maroun J., Price T., Lim R., Van Cutsem E., Park Y.S. (2007). Phase III trial of capecitabine plus oxaliplatin as adjuvant therapy for stage III colon cancer: A planned safety analysis in 1864 patients. J. Clin. Oncol..

[46] Gebremedhn E.G., Shortland P.J., Mahns D.A. (2018). The incidence of acute oxaliplatin-induced neuropathy and its impact on treatment in the first cycle: A systematic review. BMC Cancer.

[47] Sun Y.N., Jusko W.J. (1998). Transit compartments versus gamma distribution function to model signal transduction processes in pharmacodynamics. J. Pharm. Sci..

[48] Simeoni M., Magni P., Cammia C., De Nicolao G., Croci V., Pesenti E., Germani M., Poggesi I., Rocchetti M. (2004). Predictive pharmacokinetic–pharmacodynamic modeling of tumor growth kinetics in xenograft models after administration of anticancer agents. Cancer Res..

[49] Friberg L.E., Henningsson A., Maas H., Nguyen L., Karlsson M.O. (2002). Model of chemotherapy-induced myelosuppression with parameter consistency across drugs. J. Clin. Oncol..

[50] Carozzi V.A., Canta A., Chiorazzi A. (2015). Chemotherapy-induced peripheral neuropathy: What do we know about mechanisms?. Neurosci. Lett..

[51] Puchalski T.A., Krzyzanski W., Blum R.A., Jusko W.J. (2001). Pharmacodynamic modeling of lansoprazole using an indirect irreversible response model. J. Clin. Pharmacol..

[52] Park S.R., Kim M.J., Nam B.H., Kim C.G., Lee J.Y., Cho S.J., Kong S.Y., Park Y.I. (2017). A randomised phase II study of continuous versus stop-and-go S-1 plus oxaliplatin following disease stabilisation in first-line chemotherapy in patients with metastatic gastric cancer. Eur. J. Cancer.

[53] Starobova H., Vetter I. (2017). Pathophysiology of Chemotherapy-Induced Peripheral Neuropathy. Front. Mol. Neurosci..

