# Supplementary material for: Semi-Mechanism-Based Pharmacokinetic-Toxicodynamic Model of Oxaliplatin-Induced Acute and Chronic Neuropathy
Source: Pharmaceutics. 2020 Feb 3;12(2):125. doi: 10.3390/pharmaceutics12020125 (PMC7076355; doi:10.3390/pharmaceutics12020125)
Supplement: Supplementary file 1 [file pharmaceutics-12-00125-s001.zip › pharmaceutics-673480-supplementary/Revised2 Figure supp.pptx]

## Slide 1
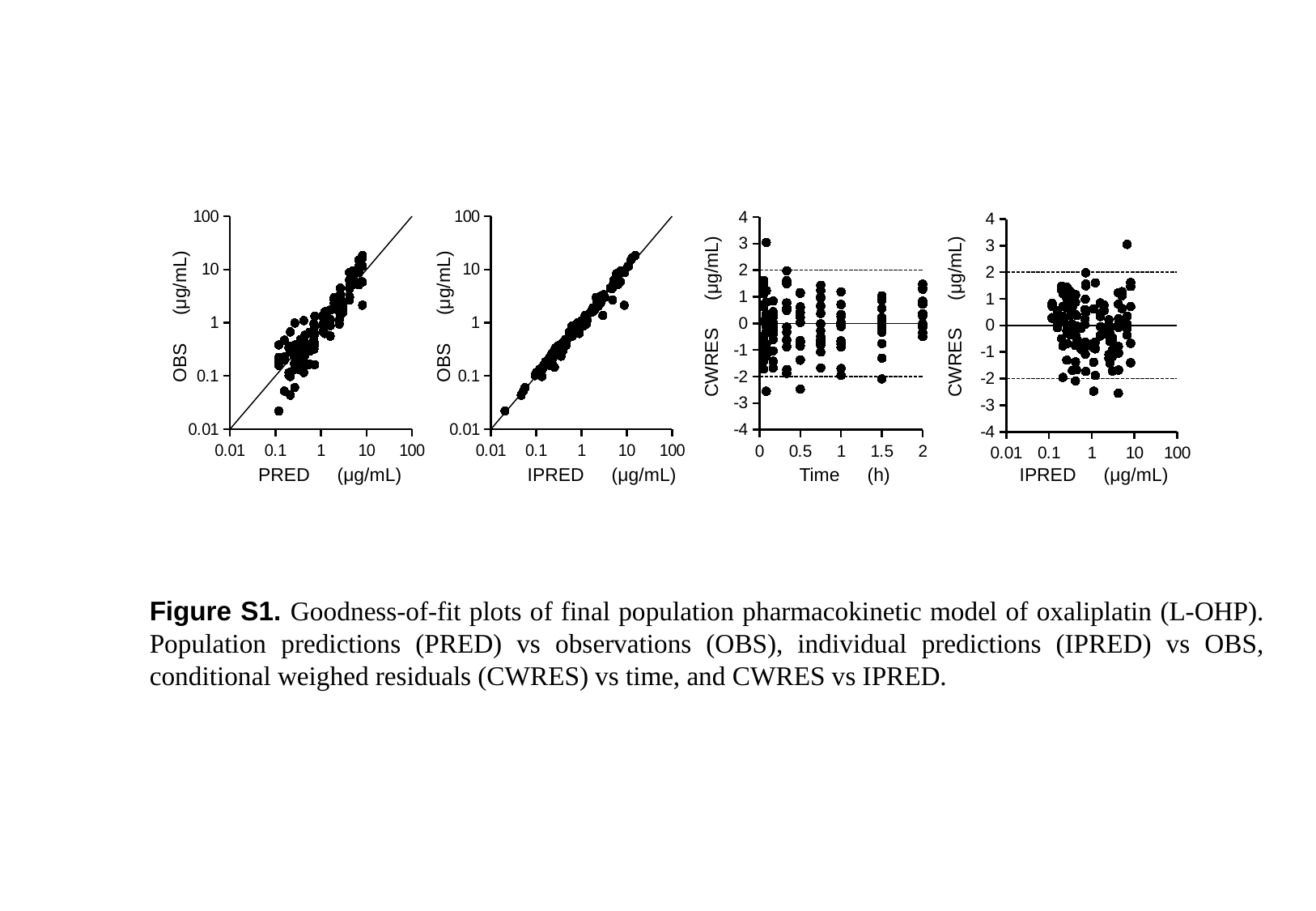

### Chart
| Category | | |
|---|---|---|
### Chart
| Category | | |
|---|---|---|
### Chart
| Category | | | | |
|---|---|---|---|---|
### Chart
| Category | | | | |
|---|---|---|---|---|CWRES　(μg/mL)
CWRES　(μg/mL)
OBS　(μg/mL)
OBS　(μg/mL)
Time　(h)
IPRED　(μg/mL)
PRED　(μg/mL)
IPRED　(μg/mL)
Figure S1. Goodness-of-fit plots of final population pharmacokinetic model of oxaliplatin (L-OHP). Population predictions (PRED) vs observations (OBS), individual predictions (IPRED) vs OBS, conditional weighed residuals (CWRES) vs time, and CWRES vs IPRED.

## Slide 2
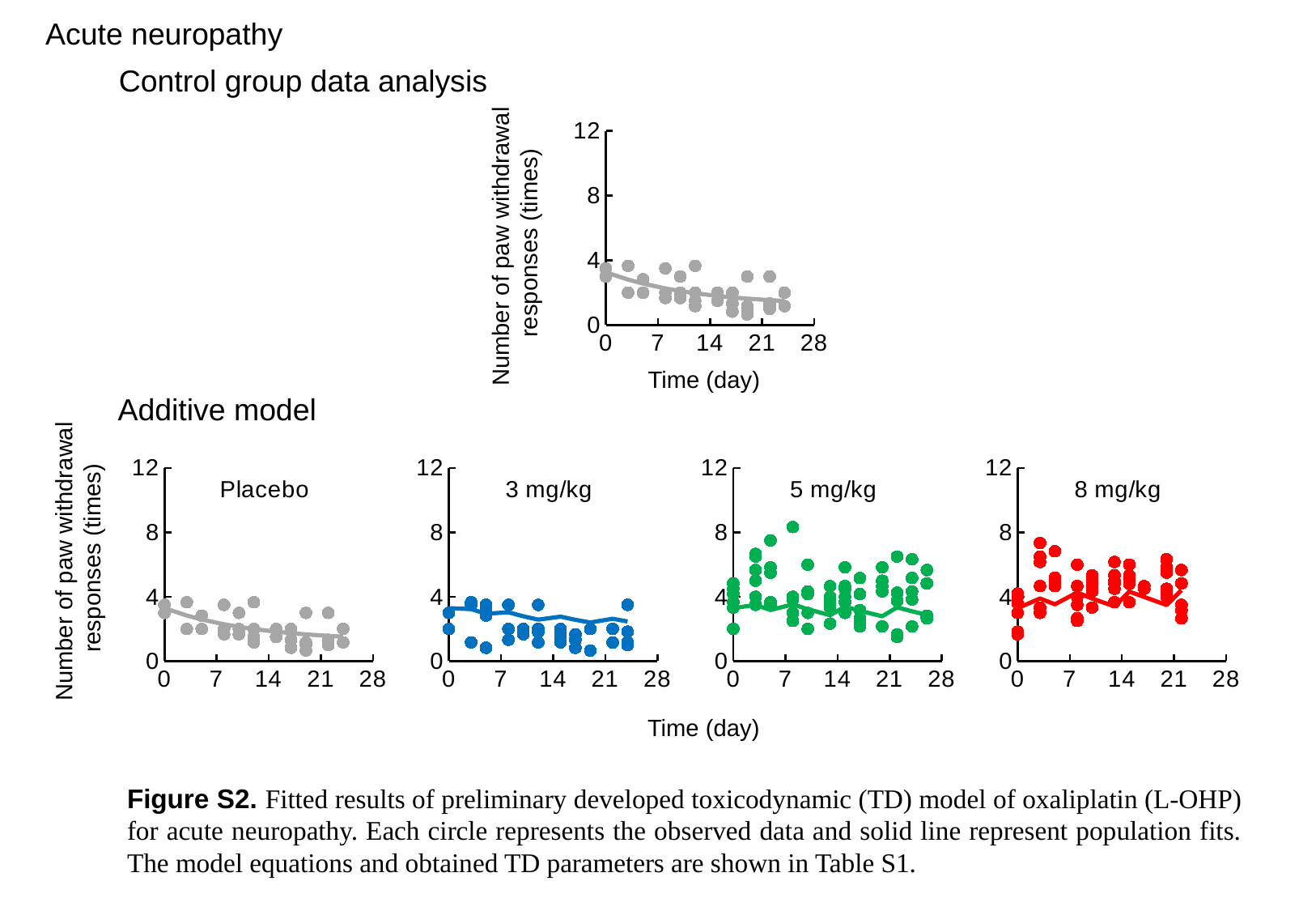

Acute neuropathy
Control group data analysis
### Chart
| Category | | |
|---|---|---|Number of paw withdrawal
 responses (times)
Time (day)
Additive model
### Chart: Placebo
| Category | | |
|---|---|---|
### Chart: 3 mg/kg
| Category | | |
|---|---|---|
### Chart: 5 mg/kg
| Category | | |
|---|---|---|
### Chart: 8 mg/kg
| Category | | |
|---|---|---|Number of paw withdrawal
 responses (times)
Time (day)
Figure S2. Fitted results of preliminary developed toxicodynamic (TD) model of oxaliplatin (L-OHP) for acute neuropathy. Each circle represents the observed data and solid line represent population fits. The model equations and obtained TD parameters are shown in Table S1.

## Slide 3
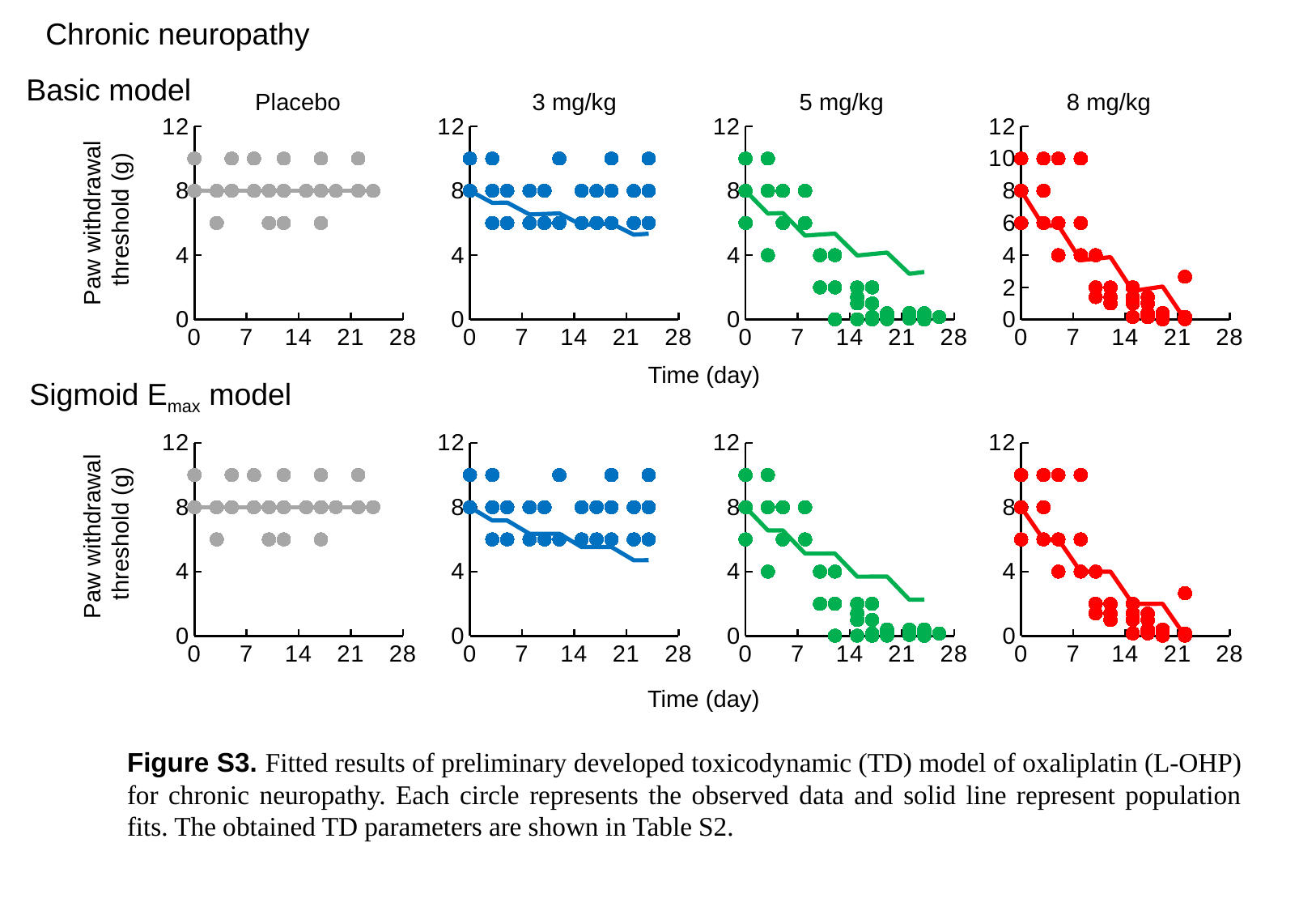

Chronic neuropathy
Basic model
Placebo
3 mg/kg
5 mg/kg
8 mg/kg
### Chart
| Category | | |
|---|---|---|
### Chart
| Category | | |
|---|---|---|
### Chart
| Category | | |
|---|---|---|
### Chart
| Category | | |
|---|---|---|Paw withdrawal
threshold (g)
Time (day)
Sigmoid Emax model
### Chart
| Category | | |
|---|---|---|
### Chart
| Category | | |
|---|---|---|
### Chart
| Category | | |
|---|---|---|
### Chart
| Category | | |
|---|---|---|Paw withdrawal
threshold (g)
Time (day)
Figure S3. Fitted results of preliminary developed toxicodynamic (TD) model of oxaliplatin (L-OHP) for chronic neuropathy. Each circle represents the observed data and solid line represent population fits. The obtained TD parameters are shown in Table S2.

## Slide 4
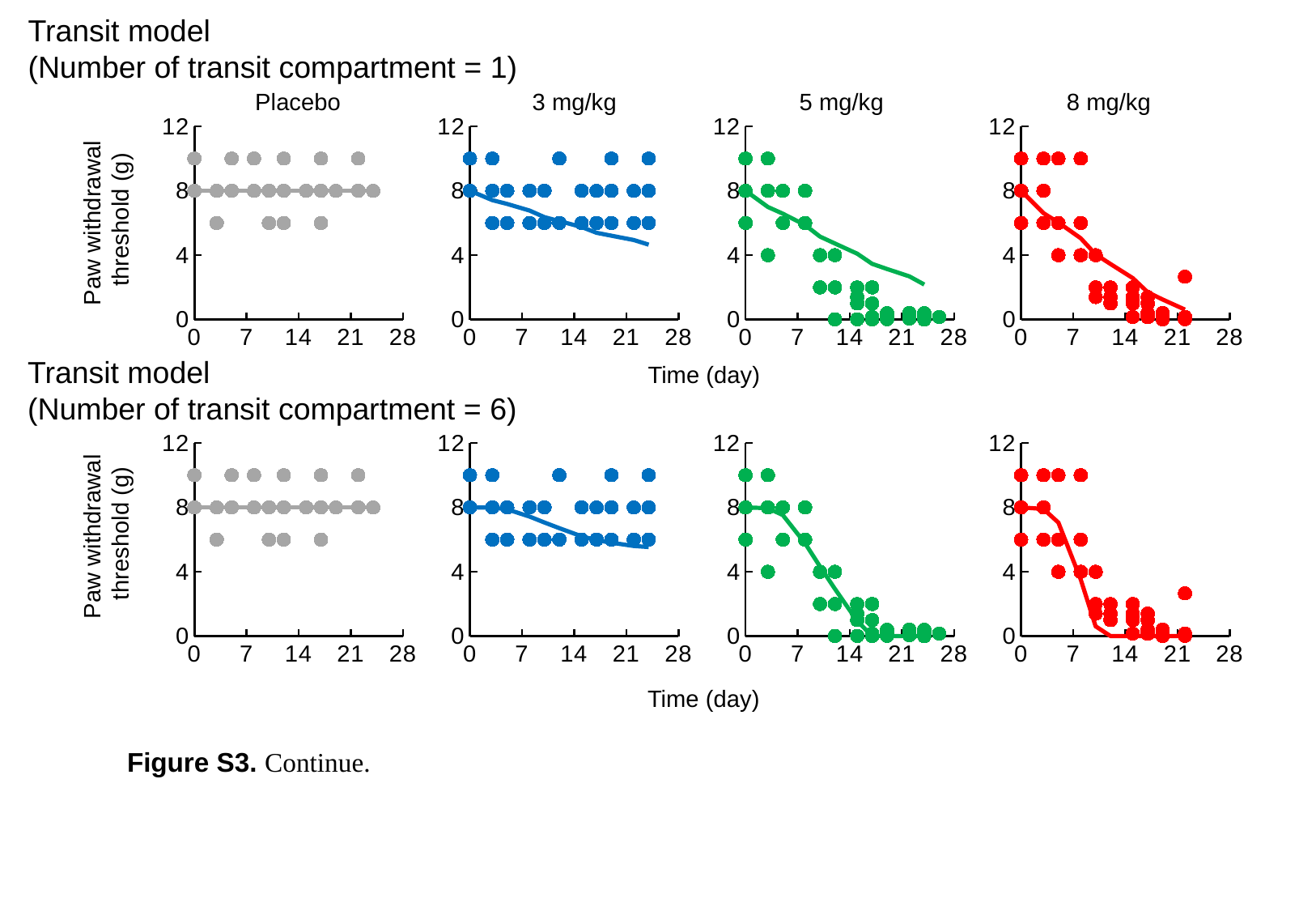

Transit model
(Number of transit compartment = 1)
Placebo
3 mg/kg
5 mg/kg
8 mg/kg
### Chart
| Category | | |
|---|---|---|
### Chart
| Category | | |
|---|---|---|
### Chart
| Category | | |
|---|---|---|
### Chart
| Category | | |
|---|---|---|Paw withdrawal
threshold (g)
Transit model
(Number of transit compartment = 6)
Time (day)
### Chart
| Category | | |
|---|---|---|
### Chart
| Category | | |
|---|---|---|
### Chart
| Category | | |
|---|---|---|
### Chart
| Category | | |
|---|---|---|Paw withdrawal
threshold (g)
Time (day)
Figure S3. Continue.

## Slide 5
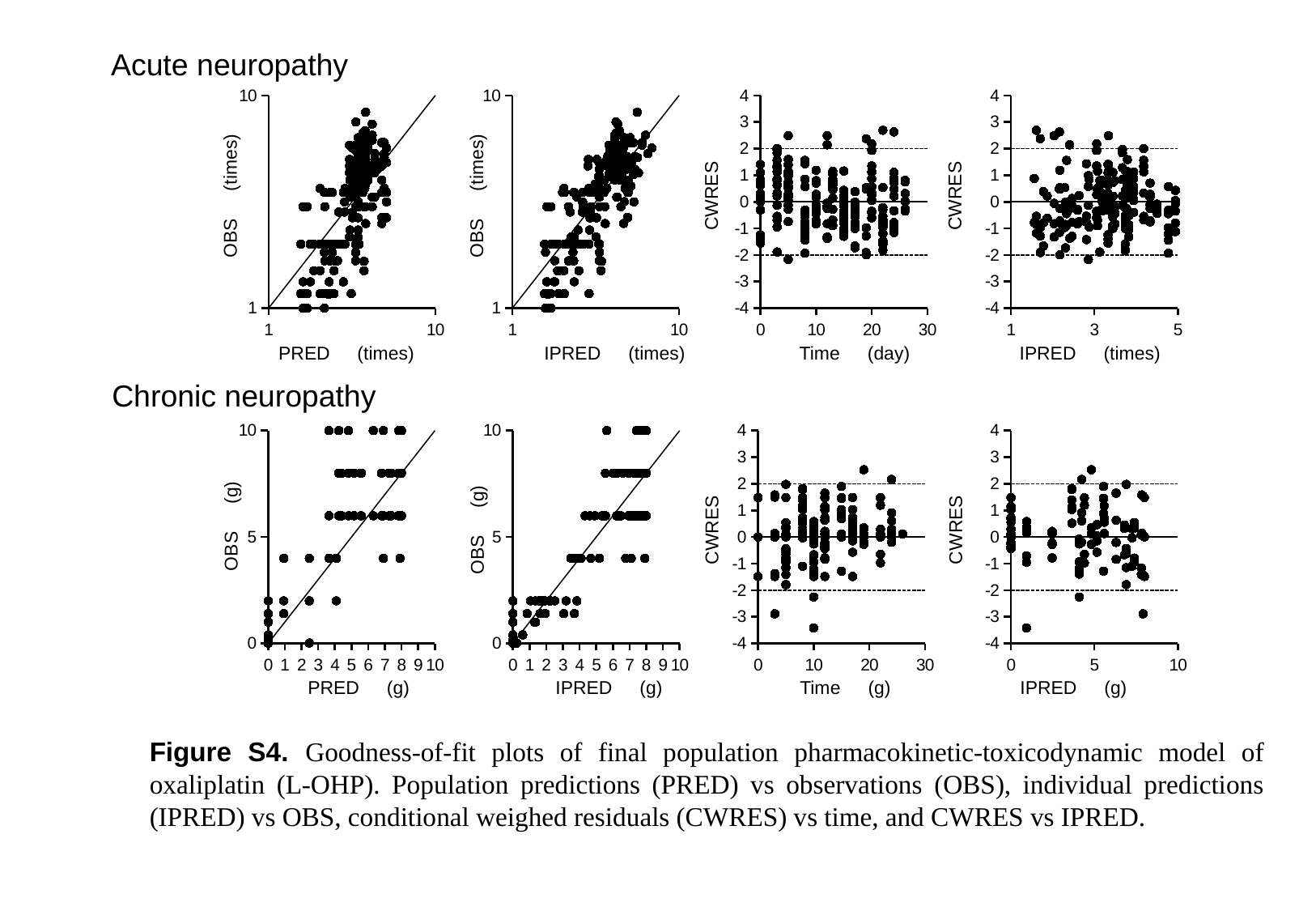

Acute neuropathy
### Chart
| Category | | |
|---|---|---|
### Chart
| Category | | |
|---|---|---|
### Chart
| Category | | | | |
|---|---|---|---|---|
### Chart
| Category | | | | |
|---|---|---|---|---|CWRES
CWRES
OBS　(times)
OBS　(times)
Time　(day)
IPRED　(times)
PRED　(times)
IPRED　(times)
Chronic neuropathy
### Chart
| Category | | |
|---|---|---|
### Chart
| Category | | |
|---|---|---|
### Chart
| Category | | | | |
|---|---|---|---|---|
### Chart
| Category | | | | |
|---|---|---|---|---|OBS　(g)
CWRES
CWRES
OBS　(g)
Time　(g)
IPRED　(g)
PRED　(g)
IPRED　(g)
Figure S4. Goodness-of-fit plots of final population pharmacokinetic-toxicodynamic model of oxaliplatin (L-OHP). Population predictions (PRED) vs observations (OBS), individual predictions (IPRED) vs OBS, conditional weighed residuals (CWRES) vs time, and CWRES vs IPRED.

## Slide 6
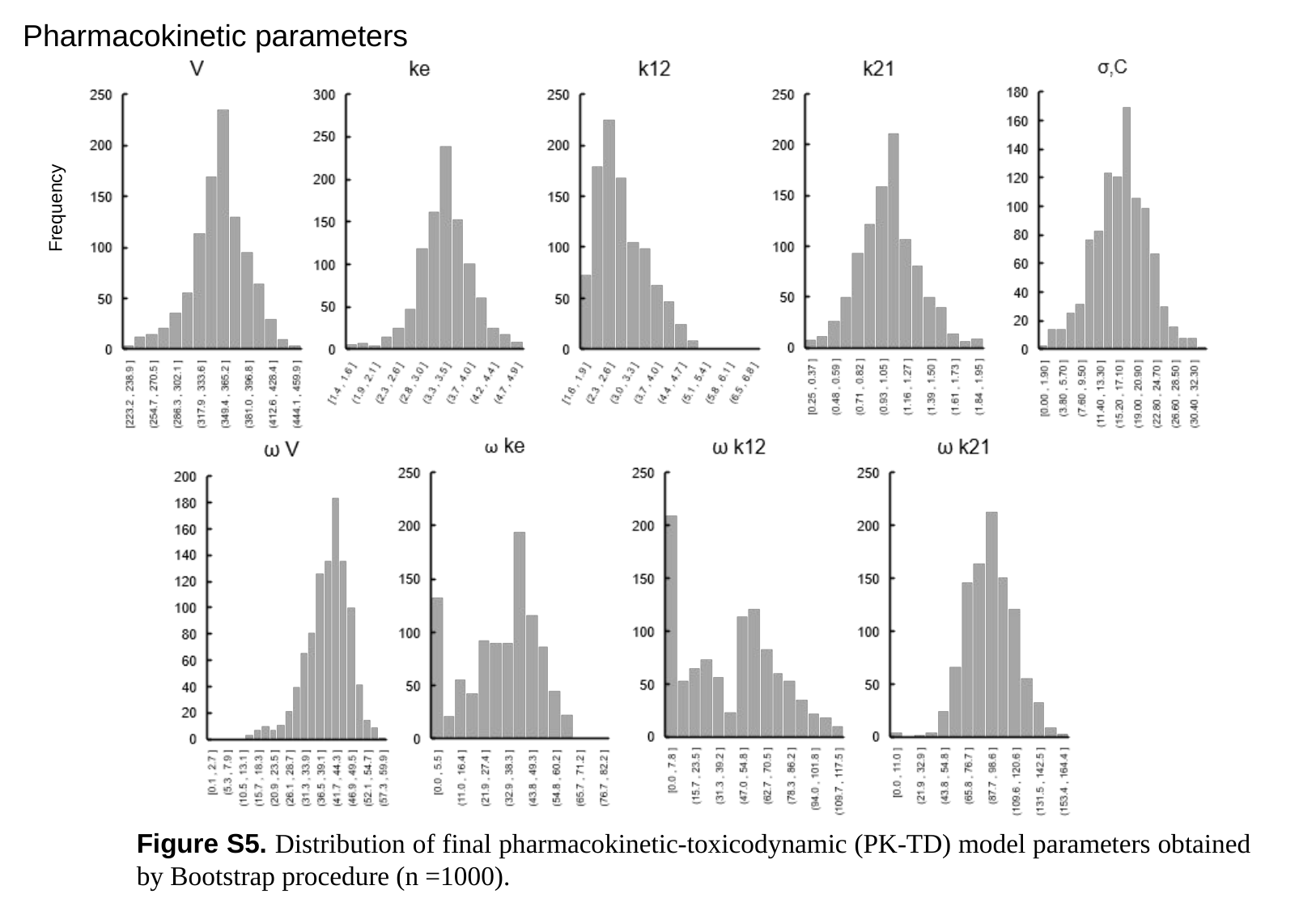

Pharmacokinetic parameters
Frequency
Figure S5. Distribution of final pharmacokinetic-toxicodynamic (PK-TD) model parameters obtained by Bootstrap procedure (n =1000).

## Slide 7
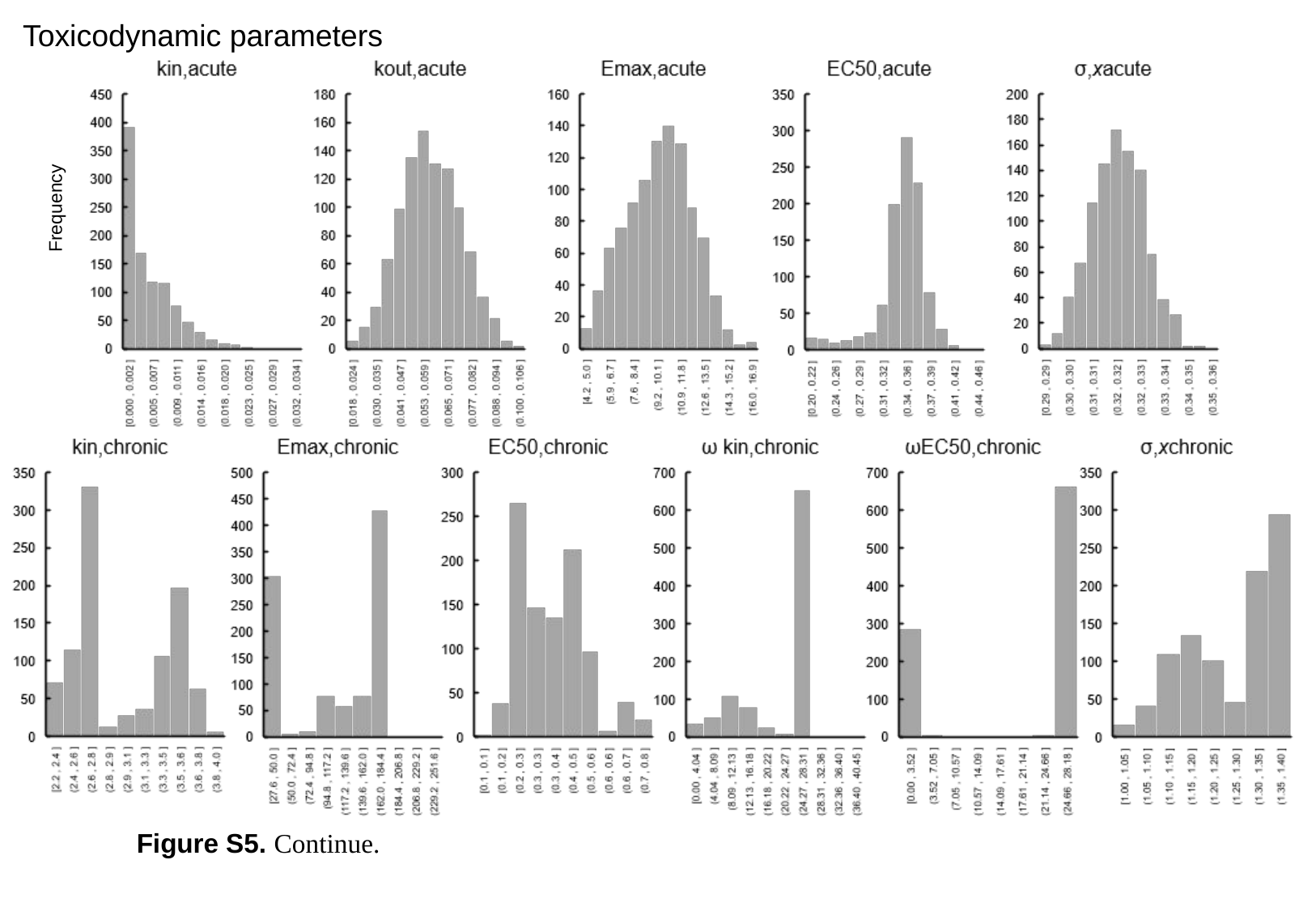

Toxicodynamic parameters
Frequency
Figure S5. Continue.
