# Supplementary material for: Semi-Mechanism-Based Pharmacokinetic-Toxicodynamic Model of Oxaliplatin-Induced Acute and Chronic Neuropathy
Source: Pharmaceutics. 2020 Feb 3;12(2):125. doi: 10.3390/pharmaceutics12020125 (PMC7076355; doi:10.3390/pharmaceutics12020125)
Supplement: Supplementary file 1 [file pharmaceutics-12-00125-s001.zip › pharmaceutics-673480-supplementary/Supp. Table .docx]

Supplementary Materials: Semi-Mechanism-Based Pharmacokinetic-Toxicodynamic Model of Oxaliplatin-Induced Acute and Chronic Neuropathy

Shinji Kobuchi, Risa Shimizu and Yukako Ito

**Table S1.** Toxicodynamic model selection for acute neuropathy.

| **Model** | **Model Equation** | **Parameters (CV %)** | **−2LL** | **AIC** |
| --- | --- | --- | --- | --- |
| Control group data analysis | $x_{acute}\left( t \right)=Response\left( t \right)+Handling(t)$,  $Handling\left( t \right)=x_{0, acute}\cdot e^{-k_{handling}\cdot t}$  $x_{acute}(0)={Handling\left( 0 \right)= x}_{0, acute}$ | *x*_0, acute_ = 3.1 (Fix) ^a^  *k_handling_ =* 0.08 (66.9) | 96.2 | 103.3 |
| Indirect response model; kout inhibition | $\frac{dResponse\left( t \right)}{dt}=k_{in, acute}-k_{out,acute}\cdot E_{L-OHP,acute}(t)\cdot Response\left( t \right)$,  $E_{L-OHP,acute}(t)=\frac{E_{max, acute}\cdot C (t)}{EC_{50,acute}+C(t)}$ | NA^b^ | 2460.9 | 2472.9 |
| Indirect response model; kin stimulate | $\frac{dResponse\left( t \right)}{dt}=k_{in, acute}\cdot E_{L-OHP,acute}(t)-k_{out,acute}\cdot Response\left( t \right)$ | NA ^b^ | 680.5 | 690.5 |
| Additive model | $\frac{dResponse\left( t \right)}{dt}=k_{in, acute}+E_{L-OHP,acute}(t)-k_{out,acute}\cdot Response\left( t \right)$ | *k*_in, acute_ = 0.002 (20.6)  *k*_out, acute_ = 0.06 (21.8)  *E*_max, acute_ = 2663 (10.0)  *EC*_50, acute_ = 315.2 (11.4) | 687.0 | 697.0 |
| Final model | $\frac{dResponse\left( t \right)}{dt}=k_{in, acute}-k_{out,acute}\cdot E_{L-OHP,acute}(t)\cdot Response\left( t \right)$,  $E_{L-OHP,acute}(t)=\frac{E_{max, acute}\cdot C^{\gamma acute}(t)}{E{C_{50,acute}}^{\gamma acute}+C^{\gamma acute}(t)}$ | See in Table 2. | 668.1 | 678.1 |

^a^ The value was derived from observed mean value. ^b^ The reliable parameter value was not available.

−2LL, −2x log likelihood; AIC, Akaike’s Information Criteria.

**Table S2.** Toxicodynamic model selection for chronic neuropathy.

| **Model** | **Model Equation** | **Parameters (CV %)** | **−2LL** | **AIC** |
| --- | --- | --- | --- | --- |
| Base model  (no transit model) | $\frac{dx_{chronic}(t)}{dt}=k_{in, chronic}\cdot(1-E_{L-OHP,chronic}(t))-k_{out,chronic}\cdot x_{chronic}(t)$, $x_{chronic}(0)=x_{0,chronic}$,  $k_{out,chronic}=k_{in,chronic}/x_{0}$,  $E_{L-OHP,chronic}(t)=\frac{E_{max, chronic}\cdot C^{\gamma chronic}(t)}{E{C_{50,chronic}}^{\gamma chronic}+C^{\gamma chronic}(t)}$ | *k*_in, chronic_ = 0.09 (0.7)  *E*_max, chronic_ = 175.4 (0.7)  *EC*_50, chronic_ =0.41 (0.7)  *γ*_chronic_ = 2 (Fix) ^a^ | 892.0 | 904.0 |
| Sigmoid E_max_ model | $E_{L-OHP,chronic}(t)=\frac{E_{max, chronic}\cdot C^{\gamma chronic}(t)}{E{C_{50,chronic}}^{\gamma chronic}+C^{\gamma chronic}(t)}$ | *k*_in, chronic_ = 0.003 (0.6)  *E*_max, chronic_ = 3158 (0.6)  *EC*_50, chronic_ =0.23 (0.6)  *γ*_chronic_ = 4 (Fix) | 878.4 | 890.4 |
| Transit model  (Number of transit compartment = 1) | $\frac{dx_{1}(t)}{dt}=k_{in, chronic}\cdot(1-E_{L-OHP,chronic}(t))-k_{out,chronic}\cdot x_{1}(t)$,  $\frac{dx_{chronic}(t)}{dt}=k_{out, chronic}\cdot x_{1}(t)-k_{out, chronic}\cdot x_{chronic}(t)$ | *k*_in, chronic_ = 0.71 (0.6)  *E*_max, chronic_ = 47.5 (34.9)  *EC*_50, chronic_ =0.23 (0.2)  *γ*_chronic_ = 4 (Fix) | 818.8 | 830.8 |
| Transit model  (Number of transit compartment = 6) | $\frac{dx_{2}(t)}{dt}=k_{out, chronic}\cdot x_{1}(t)-k_{out, chronic}\cdot x_{2}(t)$,  $\frac{dx_{n}(t)}{dt}=k_{out, chronic}\cdot x_{n-1}(t)-k_{out, chronic}\cdot x_{n}(t)$, *n* = 3, …, 6,  $\frac{dx_{chronic}(t)}{dt}=k_{out, chronic}\cdot x_{6}(t)-k_{out, chronic}\cdot x_{chronic}(t)$ | *k*_in, chronic_ = 3.5 (0.7)  *E*_max, chronic_ = 158 (0.7)  *EC*_50, chronic_ =0.50 (0.7)  *γ*_chronic_ = 4 (Fix) | 797.7 | 809.8 |
| Final model | $\frac{dx_{n}(t)}{dt}=k_{out, chronic}\cdot x_{n-1}(t)-k_{out, chronic}\cdot x_{n}(t)$, *n* = 3, …, 5,  $\frac{dx_{chronic}(t)}{dt}=k_{out, chronic}\cdot x_{5}(t)-k_{out, chronic}\cdot x_{chronic}(t)$, | See in Table 2. | 794.8 | 806.8 |

^a^ The value was fixed to obtain reliable parameter values.
